# Lp-PLA_2_ activity is associated with increased risk of diabetic retinopathy: a longitudinal disease progression study

**DOI:** 10.1007/s00125-018-4601-7

**Published:** 2018-04-06

**Authors:** Moneeza K. Siddiqui, Gwen Kennedy, Fiona Carr, Alexander S. F. Doney, Ewan R. Pearson, Andrew D. Morris, Toby Johnson, Megan M. McLaughlin, Rachel E. Williams, Colin N. A. Palmer

**Affiliations:** 1 Pat McPherson Centre for Pharmacogenetics and Pharmacogenomics, University of Dundee School of Medicine, Ninewells Hospital and Medical School, Dundee, DD1 9SY UK; 2 Ninewells Hospital and Medical School, University of Dundee, Dundee, UK; 3 Usher Institute of Population Health Sciences and Informatics, University of Edinburgh, Edinburgh, UK; 4 GlaxoSmithKline Medicines Research Centre, Gunnels Wood Road, Stevenage, UK; 5 Alternative Discovery and Development, Research & Development, GlaxoSmithKline, King of Prussia, PA USA; 6 Worldwide Epidemiology, GlaxoSmithKline, Collegeville, PA USA

**Keywords:** Diabetic complications, Electronic medical records, Epidemiology, Lipids/lipoproteins, Microvascular disease, Retinopathy

## Abstract

**Aims/hypothesis:**

The aim of the study was to examine the association between lipoprotein-associated phospholipase A_2_ (Lp-PLA_2_) activity levels and incident diabetic retinopathy and change in retinopathy grade.

**Methods:**

This was a cohort study of diabetic participants with serum collected at baseline and routinely collected diabetic retinal screening data. Participants with type 2 diabetes from the GoDARTS (Genetics of Diabetes Audit and Research in Tayside Scotland) cohort were used. This cohort is composed of individuals of white Scottish ancestry from the Tayside region of Scotland. Survival analysis accounting for informative censoring by modelling death as a competing risk was performed for the development of incident diabetic retinopathy from a disease-free state in a 3 year follow-up period (*n* = 1364) by stratified Lp-PLA_2_ activity levels (in quartiles). The same analysis was performed for transitions to more severe grades.

**Results:**

The hazard of developing incident diabetic retinopathy was 2.08 times higher (95% CI 1.64, 2.63) for the highest quartile of Lp-PLA_2_ activity compared with the lowest. Higher Lp-PLA_2_ activity levels were associated with a significantly increased risk for transitions to all grades. The hazards of developing observable (or more severe) and referable (or more severe) retinopathy were 2.82 (95% CI 1.71, 4.65) and 1.87 (95% CI 1.26, 2.77) times higher for the highest quartile of Lp-PLA_2_ activity compared with the lowest, respectively.

**Conclusions/interpretation:**

Higher Lp-PLA_2_ levels are associated with increased risk of death and the development of incident diabetic retinopathy, as well as transitions to more severe grades of diabetic retinopathy. These associations are independent of calculated LDL-cholesterol and other traditional risk factors. Further, this biomarker study shows that the association is temporally sensitive to the proximity of the event to measurement of Lp-PLA_2._

**Electronic supplementary material:**

The online version of this article (10.1007/s00125-018-4601-7) contains peer-reviewed but unedited supplementary material, which is available to authorised users.

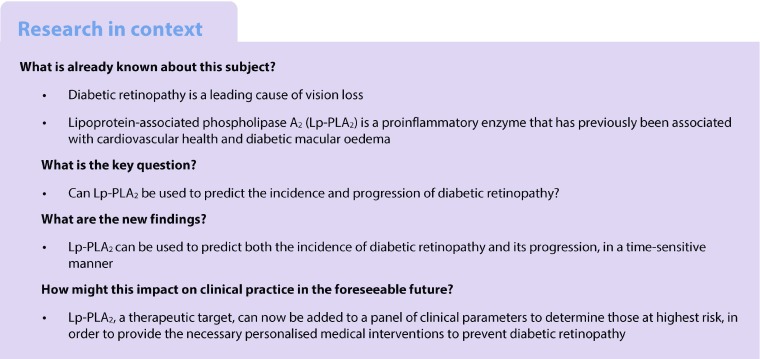



## Introduction

Diabetic retinopathy is a leading cause of vision loss and blindness in the working age population (20–74 years of age) of most developed countries [1]. It was found to occur in 35% of people with diabetes based on a meta-analysis of multiple studies [2]. The increasing number of individuals with diabetes worldwide suggests that diabetic retinopathy is likely to be a growing contributor to vision loss and associated functional impairment in the future [3].

Risk factors associated with diabetic retinopathy include age, race/ethnicity, longer duration of diabetes, insulin dependence, younger age of diabetes onset, higher HbA_1c_, insulin treatment and higher blood pressure [4–10]. Studies have also shown that hyperlipidaemia, hyperglycaemia and hypertension contribute to the pathogenesis of diabetic retinopathy [11–14]. Smoking is generally not considered a risk factor; however, at least one study found a significant association between smoking and diabetic macular oedema (DME), a condition that progresses from retinopathy, in people with type 1 diabetes [15]. For decades, the management paradigm for diabetic retinopathy and DME had been early detection, optimal glycaemic control, blood pressure control, laser photocoagulation and surgery, if necessary [3, 16, 17]. More recently, intravitreally administered medications such as anti-vascular endothelial growth factor (VEGF) agents and corticosteroids have shown beneficial effects [16, 18–20]. However, a number of high-risk participants are not identified by current methods of screening [3].

Lipoprotein-associated phospholipase A_2_ (Lp-PLA_2_) is a vascular-specific, proinflammatory enzyme that binds to plasma lipoproteins (~70–80% to LDL-cholesterol [LDLc], the rest to HDL-cholesterol). Packard et al first demonstrated, in the West of Scotland Coronary Prevention Study (WOSCOPS) trial, that Lp-PLA_2_ activity is associated with increased risk of coronary events [21]. Subsequent studies have confirmed that Lp-PLA_2_ activity is prospectively associated with increased risk of coronary heart disease, independent from risk attributable to circulating lipid levels [22, 23].

Lp-PLA_2_ has been postulated to play an important role in diabetes-induced vascular leakage, which may cause the breakdown of the inner blood–retinal barrier that is observed in early and more advanced stages of retinopathy and DME. Studies with Lp-PLA_2_ inhibitors, darapladib and SB-435495 (GlaxoSmithKline, King of Prussia, PA, USA), have provided evidence of reduced leakage across the blood–retinal barrier in diabetic participants and a rat model of diabetes, respectively [24, 25]. In addition, darapladib has been shown to reduce leakage across the blood–brain barrier in diabetic and hypercholesteraemic pig models [26].

The purpose of this study is to explore whether variation in Lp-PLA_2_ activity measured in serum samples from a diabetic population is associated with subsequent incidence of, or progression from less to more severe, retinopathy.

## Methods

### Basic information about the study cohort

Diabetes Audit and Research in Tayside Scotland (DARTS) is an integrated clinical management system linking all clinical events of individuals with type 2 diabetes in the Tayside region of Scotland and provides complete continuity of care from general physicians, diabetes specialists and population screening services. GoDARTS (Genetics of DARTS) is a sub-cohort study of individuals from DARTS who have provided a sample of blood for genetic studies and have given consent for linking genetic data to study complications of diabetes, the clinical data for which is continually updated from the electronic medical record [27]. Between 2004 and 2007, participants with type 2 diabetes were recruited (together with non-diabetic controls, who were not included in this study). A serum sample was also collected from every participant at recruitment, and a series of characteristics was measured. Recruitment was treated as the baseline for this study. A cohort study in GoDARTS with a 3 year follow-up period was used to evaluate the primary objective: to test the association between Lp-PLA_2_ activity levels (measured at baseline) and incident diagnosis of retinopathy, as well as progression of retinopathy grade.

### Measurement of exposure: Lp-PLA_2_ activity

The exposure of interest was Lp-PLA_2_ activity level, measured in serum samples taken at baseline. The biobanked serum samples for this population were analysed for Lp-PLA_2_ activity using the CAM colorimetric assay (diaDexus, San Francisco, CA, USA) [28]. Note that Lp-PLA_2_ mass was neither measured nor analysed. The activity assay was performed at the Immunoassay Biomarker Core Laboratory, University of Dundee. The diaDexus assay was provided directly from the manufacturer to the laboratory. The intra-assay %CV (*n* = 36) was 5.8%. The inter-assay (assay to assay) %CV (*n* = 8) was 7.6%. The duplicate intra-assay %CV of those participants measured in duplicate (*n* = 134) was 3.0% (range 0%–18.6%).

### Measurement of main outcome: diabetic retinopathy

For retinopathy, data were acquired from the Scottish National Diabetic Eye Screening Service. The severity of retinopathy was coded as per the Scottish Diabetes Retinal Grading Scheme (www.ndrs-wp.scot.nhs.uk/wp-content/uploads/2013/04/Grading-Scheme-2007-v1.1.pdf), and are denoted as follows: disease-free (DR0), mild (DR1), observable (DR2), referable (DR3) and proliferative (DR4). Retinal screening in Tayside, Scotland has been undertaken since 1990, initially with Polaroid images, progressing to digital images in 2000 [10]. The Tayside diabetic retinopathy screening protocol has been described previously [29] and the GoDARTS cohort has been used previously by Liu et al to examine risk factors for the progression of diabetic retinopathy [10]. This cohort provides a high-resolution longitudinal dataset for the study of retinal disease progression. Data includes stages of retinopathy, recorded separately for both eyes, and dates. The eye with more advanced retinopathy stage was used for analysis. The screening data used in this study are from the years 1990 to 2011. The most recent screening data prior to baseline was used to establish the prevalent retinopathy stage. The primary outcome variables were time to first occurrence in the record of DR1, DR2, DR3 or DR4 (i.e. incident retinopathy). Time to DR2 was assessed as the time from baseline until first visit where grade DR2 was recorded, and similarly for times to DR3 or DR4. However, this study does not examine the association of Lp-PLA_2_ with DME, as the screening data does not contain information specific to DME status.

### Competing risk: association of Lp-PLA_2_ with death in GoDARTS

Previous studies of diabetic and non-diabetic participants have demonstrated that Lp-PLA_2_ activity levels are strongly associated with incident coronary heart disease and mortality [23]. In GoDARTS, death during the follow-up period was determined from the administrative records of the General Registrar Office, which holds details of deaths throughout Scotland. Time from baseline to death (for the full cohort) was analysed using the Cox proportional hazards model, with age and sex as covariates, and with quartiles of baseline Lp-PLA_2_ activity levels as the exposure (see electronic supplementary material [ESM] Table 1 and ESM Fig. 1).

### Exclusion and inclusion criteria

Exploratory analyses suggested that the modelling assumptions were sensitive to the temporal proximity of death and retinopathy events to the time of serum collection and Lp-PLA_2_ measurement. Therefore, a follow-up period of 3 years was chosen to reduce the potential for bias, while maintaining follow-up for enough incident and progression events to accrue. The main analyses reported considered only outcomes that occurred within a 3 year follow-up period after baseline for each participant. Participants with no diabetic retinopathy assessment, and who also did not die within this follow-up period, were excluded completely from the main analyses. The effect of these exclusions (which made the main analysis sample size substantially smaller than the full cohort size) was assessed using sensitivity analyses. Since participants with DR4 at baseline could not be analysed for further disease progression, these participants were also excluded from all analyses. Of the full cohort of *n* = 6731 participants, *n* = 684 were excluded because they had DR4 at baseline (Table 1), a further *n* = 4667 were excluded from the main analyses because they had inadequate follow-up (neither death nor retinopathy assessment within 3 years), and finally 16 individuals were excluded from the analyses as they had inadequate baseline covariate information. Hence 1364 participants were included in the study cohort for analyses.

**Table 1:** Baseline demographics of the full GoDARTS cohort (*n* = 6731) and study cohort (*n* = 1364)

Variable	Baseline population (*n* = 6731)		Study cohort (*n* = 1364)	
		Association with Lp-PLA_2_		Association with Lp-PLA_2_
		β estimate (95% CI)		β estimate (95% CI)
Lp-PLA_2_, nmol min^−1^ ml^−1^	121.2 ± 34.8	–	113.4 ± 32.5	–
Age, years	65 ± 11	−0.41 (−0.48, −0.34)	67 ± 11	−0.38 (−0.45,−0.30)***
Sex (% women)	44	−13.87 (−15.51, −12.23)***	41	−14.00 (−15.51, −12.23)***
Smokers (% ever smokers)	62	4.33 (2.61, 6.06)*	63	4.33 (2.61, 6.05)***
Statin users (% statin users)	90	−9.42 (−12.00, −6.85)***	91	−9.63 (−12.60, −6.57)***
Diabetes-controlling medication users (% users)	75	−4.58 (−6.40, −2.75)	81	−4.81 (−6.77, −2.86)**
Duration of diabetes, years	7 ± 6	−0.001 (−0.0015, −0.0007)	9 ± 7	−0.002 (−0.0015, −0.0007)***
BMI, kg/m^2^	31 ± 6	−0.12 (−0.25, −0.98)*	31 ± 6	−0.16 (−0.30, −0.02)***
Baseline clinical data				
Calculated LDLc, mmol/l	2.09 ± 0.77	19.70 (18.72, 20.67)***	2.10 ± 0.80	20.00 (18.54, 20.57)***
HbA_1c_, mmol/mol	58 ± 16	0.17 (0.12, 0.22)***	59 ± 18	0.16 (0.11, 0.22)***
HbA_1c_, %	7.5 ± 1.45	1.83 (1.26, 2.40)***	7.60 ± 1.60	1.83 (1.26, 2.40)***
SBP, mmHg	142 ± 19	−0.07 (−0.11, −0.30)*	143 ± 20	−0.08 (−0.12, −0.03)
DBP, mmHg	77 ± 12	0.28 (0.21, 0.35)***	75 ± 12	0.27 (0.20, 0.34)***
eGFR, ml min^−1^ [1.73 m]^−2^	85 ± 20	0.05 (0.005, 0.099)*	85 ± 20	0.05 (0.006, 0.1)*
Baseline diabetic retinopathy status (reference group DR0)				
DR0: no diabetic retinopathy, *n* (%)	3207 (50.30)	−1.44 (−2.10, −0.80)***	548 (40.20)	−1.44 (−2.09, −0.80)***
DR1: mild, *n* (%)	1496 (23.50)		464 (34.00)	
DR2: moderate, *n* (%)	968 (15.20)		345 (25.30)	
DR3: severe, *n* (%)	16 (0.30)		7 (0.50)	
DR4: proliferative, *n* (%)	684 (10.70)		–	

Data are mean ± SD, % or *n* (%) Associations were tested using univariate linear regression, with Lp-PLA_2_ as the outcome

***p < 0.001, **p < 0.01, *p < 0.05

### Statistical methods

Lp-PLA_2_ measurements were used as a linear trait for association testing with baseline covariates and divided into quartiles for cumulative incidence modelling of survival analysis. Means and SDs of Lp-PLA_2_ activity are presented. Diabetic retinopathy grades were the outcome event and were treated as a binary (yes or no) variable for survival analyses. Baseline variables were selected for potential inclusion as covariates, based on known associations with diabetic retinopathy. Variables considered as potential covariates in the analyses were an individual’s retinopathy status prior to baseline (estimated from the last visit at or prior to serum collection date), and participant age, sex, prior duration of diabetes, BMI, calculated LDLc, HbA_1c_, smoking status, systolic and diastolic blood pressure (SBP and DBP), eGFR, use of statins and use of diabetes-controlling medication (all measured at baseline). The association with baseline covariates was assessed using univariate linear regression. Variables found to be significantly associated in univariate regressions were then tested in survival analyses, and non-significant variables were eliminated from the final model. Survival analyses adjusted for competing risk of death were conducted with Lp-PLA_2_ activity levels (in quartiles) using a cumulative incidence model. Results of survival analyses are presented as the omnibus test for the Lp-PLA_2_ variable, HR and 95% CI. The results of the main effects (unadjusted) and final (adjusted) models on Lp-PLA_2_ hazards are shown in Table 2. Individuals with missing data were excluded on a per-analysis basis.

**Table 2:** Association of predictors and covariates included in each analysis

Variable	HR	95% CI	*p* value
Development of incident retinopathy from disease-free state^a^			
Main effects model			
Lp-PLA_2_ Q2 vs Q1	1.33	1.08, 1.64	<0.01
Lp-PLA_2_ Q3 vs Q1	1.56	1.28, 1.90	<0.0001
Lp-PLA_2_ Q4 vs Q1	1.52	1.24, 1.86	<0.0001
Final model^b^			
Lp-PLA_2_ Q2 vs Q1	1.52	1.21, 1.91	<0.001
Lp-PLA_2_ Q3 vs Q1	1.72	1.37, 2.17	<0.001
Lp-PLA_2_ Q4 vs Q1	2.08	1.64, 2.63	<0.001
Progression to observable or more severe retinopathy from lower grades^c^			
Main effects model			
Lp-PLA_2_ Q2 vs Q1	1.86	1.29, 2.77	<0.01
Lp-PLA_2_ Q3 vs Q1	2.33	1.59, 3.42	<0.0001
Lp-PLA_2_ Q4 vs Q1	2.21	1.46, 3.331	<0.001
Final model^d^			
Lp-PLA_2_ Q2 vs Q1	1.96	1.23, 3.00	<0.01
Lp-PLA_2_ Q3 vs Q1	2.71	1.75, 4.20	<0.001
Lp-PLA_2_ Q4 vs Q1	2.82	1.71, 4.65	<0.001
Progression to referable or proliferative retinopathy from lower grades^e^			
Main effects model			
Lp-PLA_2_ Q2 vs Q1	1.76	1.23, 2.50	<0.01
Lp-PLA_2_ Q3 vs Q1	1.81	1.26, 2.60	<0.01
Lp-PLA_2_ Q4 vs Q1	1.83	1.25, 2.70	<0.01
Final model^f^			
Lp-PLA_2_ Q2 vs Q1	1.64	1.13, 2.37	<0.01
Lp-PLA_2_ Q3 vs Q1	1.98	1.34, 2.92	<0.01
Lp-PLA_2_ Q4 vs Q1	1.87	1.26, 2.77	<0.01

^a^DR0 to DR1 or higher, *n* = 1013

^b^Lp-PLA_2_ omnibus test for variable: Wald *χ*^2^, 38.2 (df = 3) *p* value = 3 × 10^−8^

^c^DR0 or DR1 to DR2 or higher, *n* = 1241

^d^Lp-PLA_2_ omnibus test for variable: Wald *χ*^2^, 23.6 (df = 3) *p* value = 1.5 × 10^−53^

^e^DR0, DR1 or DR2 to DR3 or higher, *n* = 1364

^f^Lp-PLA_2_ omnibus test for variable: Wald *χ*^2^, 16.8 (df = 3) *p* value = 2.8 × 10^−3^

Q, quartile

The cumulative incidence graphs presented are unadjusted, and demonstrate the main effect of Lp-PLA_2_ quartiles on the development and progression of retinopathy. All HRs of Lp-PLA_2_ use the lowest quartile of Lp-PLA_2_ activity as the reference. All statistical analyses were conducted using SAS 9.4 (SAS institute, Cary, NC, USA).

### Ethics approval and participant consent

The Tayside Medical Ethics Committee has approved the GoDARTS study and informed consent was obtained for all participants. The participants have consented to research on their samples and data related to diabetes, its treatment and related conditions. They have also consented to the fact that the studies may involve collaborative studies with commercial companies and that the participants will not benefit financially from such collaborations. Neither the University of Dundee research team nor GlaxoSmithKline had access to personally identifiable information. All event level data is provided to the University of Dundee research team in anonymised fashion by the Data Linkage team of the Health Informatics Centre at the University of Dundee.

## Results

### Baseline associations

**Fig. 1:**
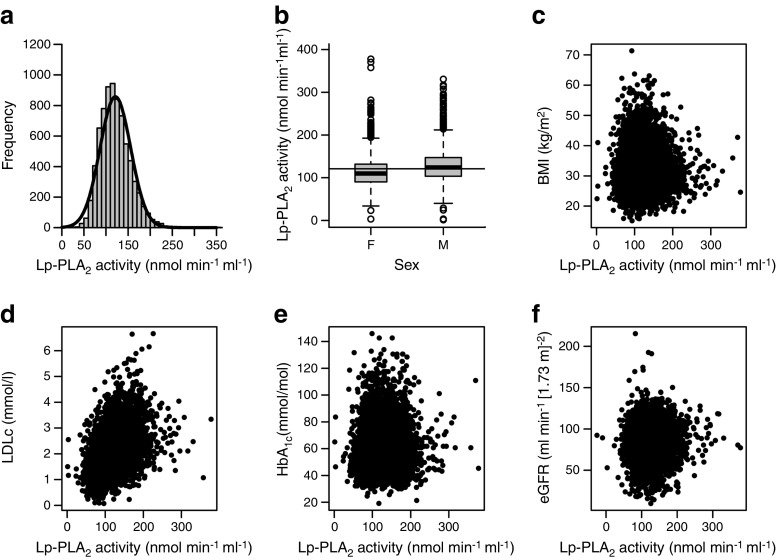
(**a**) Baseline distribution of Lp-PLA_2_ activity (nmol min^−1^ ml^−1^). (**b**) Distribution by sex (β = −0.12, *p* < 0.0001). (**c**) Correlation with BMI (kg/m^2^) (*r* = −0.02, *p* = 0.07). (**d**) Correlation with LDLc (mmol/l) (*r* = 0.44, *p* < 0.0001). (**e**) Correlation with HbA_1c_ (mmol/mol) (*r* = 0.08, *p* = 0.0003). (**f**) Correlation with eGFR (ml min^−1^ [1.73 m]^−2^) (*r* = 0.03, *p* = 0.026). F, female; M, male

All diabetic participants in GoDARTS with available serum were assayed for Lp-PLA_2_ activity. Measured Lp-PLA_2_ activity levels were approximately normally distributed (presented in Fig. 1a) and were therefore analysed as an untransformed, continuous variable for baseline analysis. The mean (SD) Lp-PLA_2_ activity level was 121 (35) nmol min^−1^ ml^−1^. The observed quartiles were Q1 ≤ 97.2, Q2 ≤ 117.3, Q3 ≤ 140.7 and Q4 ≤ 377.9 nmol min^−1^ ml^−1^, which were used to divide the participants into equal sized groups for survival analyses.

Their sex, age, usage of diabetes-controlling medication, usage of statins, smoking status, duration of diabetes, HbA_1c_, LDLc levels, eGFR and SBP were all associated with their Lp-PLA_2_ activity levels. The distribution of Lp-PLA_2_, and the association of sex, BMI, LDLc and HbA_1c_ with Lp-PLA_2_ in the population is presented in Fig. 1. Lp-PLA_2_ levels stratified by sex showed significantly different levels; women have lower levels than men (Fig. 1b). The mean (SD) Lp-PLA_2_ levels amongst women was 114 (35) nmol min^−1^ ml^−1^ compared with 127 (35) nmol min^−1^ ml^−1^ for men. The correlation of Lp-PLA_2_ activity levels with BMI, LDLc, HbA_1c_ and eGFR at baseline are also shown in Fig. 1c–f, respectively. The only notable correlation was with LDLc, which showed a strong linear relationship with Lp-PLA_2_, with a correlation coefficient of 0.44.

The association of these potentially confounding variables with Lp-PLA_2_ is presented for the study population and the full GoDARTS cohort (Table 1). Variables showed similar associations in the study population compared with the full GoDARTS cohort; however, the study cohort were on average 2 years older, had type 2 diabetes for 2 years longer, a higher proportion were medicated for type 2 diabetes, and a lower proportion were disease-free (DR0) at baseline. This suggests that the study cohort had more severe disease at baseline and were therefore more likely to be screened regularly for progression during follow-up.

### Association of Lp-PLA_2_ with death

In agreement with results from previous large meta-analyses including both diabetic and non-diabetic participants [23], Lp-PLA_2_ activity levels were strongly associated with death in the diabetic population of the GoDARTS cohort for whom Lp-PLA_2_ activity was measured (*n* = 6731). This is demonstrated in ESM Fig. 1 and ESM Table 1 as an increased risk of death for participants in the highest two quartiles of Lp-PLA_2_ levels compared with the lowest. The hazard of death for those in the highest Lp-PLA_2_ quartile was approximately one and a half times that for participants in the lowest quartile (HR 1.45, 95% CI 1.24, 1.68; *p* < 0.001). Hence, when analysing the association between Lp-PLA_2_ activity levels and time to progression, censoring at death would violate the non-informative censoring assumption required for standard survival analyses such as Cox regression [30]. Therefore, a competing risk survival analysis [31] was used to analyse the association with incident diabetic retinopathy and progression to more severe stages.

### Survival analyses

#### Association of Lp-PLA_2_ activity with incident diabetic retinopathy

For this analysis, a cohort of 1013 individuals who had no observable retinopathy (DR0) at baseline were included. Of these, 676 individuals progressed to any retinopathy grade (mild retinopathy: DR1, or higher) in the 3 year follow-up period. In the same cohort there were 143 deaths prior to any record of progression, and 194 individuals who were censored (alive and without progression).

As shown in Table 2, there was a progressive trend of increased risk across the quartiles. The hazards of developing incident retinopathy were 1.52, 1.72 and 2.08 for Lp-PLA_2_ quartiles 2, 3 and 4, respectively, compared with quartile 1. The omnibus test of hazard across the quartiles of Lp-PLA_2_ activity was highly significant (*p* = 3 × 10^−8^). This analysis was adjusted for sex, diabetes-controlling medication, use of statins, HbA_1c_ levels, systolic blood pressure, LDLc levels and the age of the individual at baseline and was therefore independent of traditional risk factors for diabetic retinopathy.

**Fig. 2:**
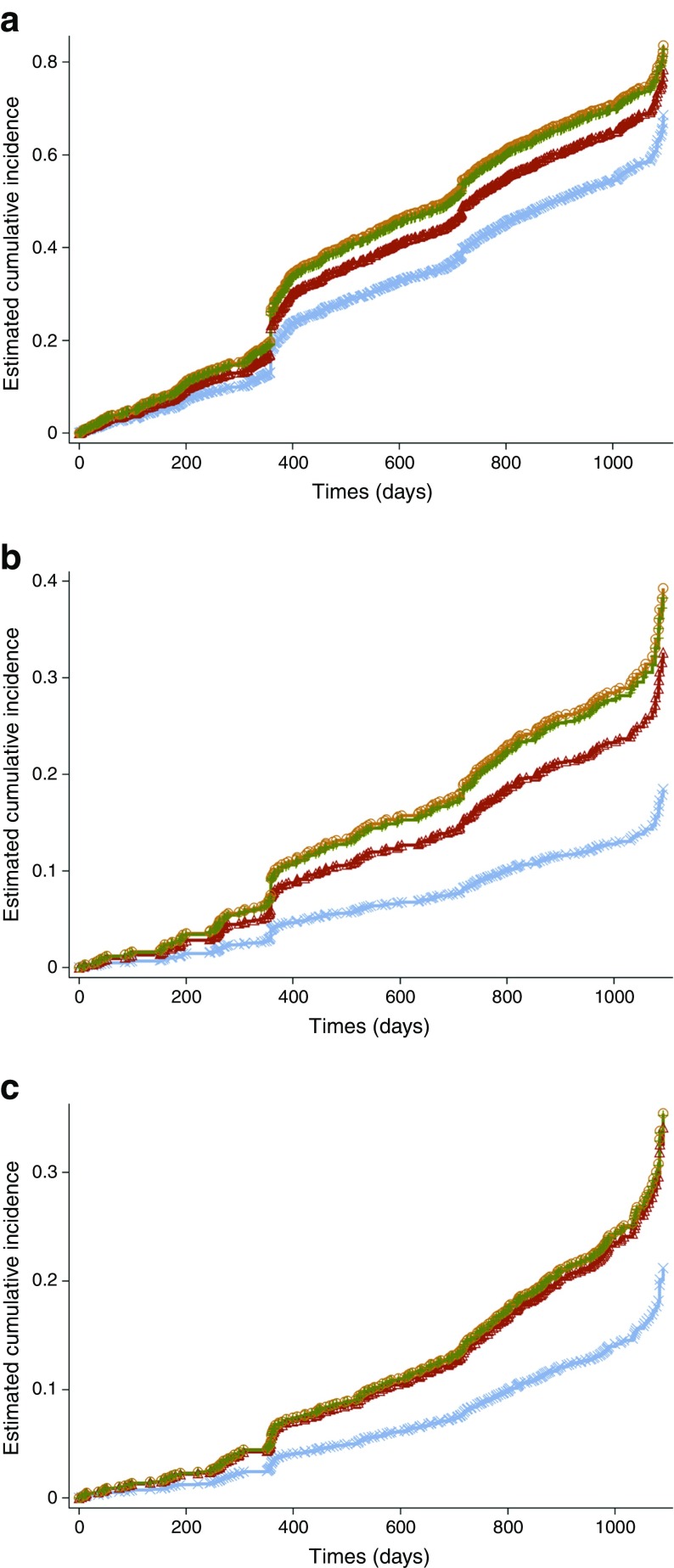
(**a**) Cumulative incidence plot of the hazards of incident diabetic retinopathy for Lp-PLA_2_ activity quartiles, main effects model. (**b**) Cumulative incidence plot of the hazards of progression to observable or more severe retinopathy by Lp-PLA_2_ activity quartiles, main effects model. (**c**) Cumulative incidence plot of hazards of progression to referable or proliferative retinopathy by Lp-PLA_2_ activity quartiles, main effects model. Light blue line with crosses, lowest quartile (quartile 1) of Lp-PLA_2_ activity; red line with triangles, quartile 2; brown line with circles, quartile 3; green line with crosses, highest quartile (quartile 4). Symbols denote events

The accompanying cumulative incidence plot (Fig. 2a) is unadjusted and shows the increased hazards of incident retinopathy for Lp-PLA_2_ activity quartiles relative to the lowest quartile (light blue line).

The GoDARTS study largely recruited at the time of the participants’ annual diabetes review, which accounts for the evident increase in retinopathy diagnosis at the 1 year mark, coinciding with the next annual review after recruitment.

#### Association of Lp-PLA_2_ activity with progression to observable or more severe diabetic retinopathy from lower grades

For this analysis, a cohort of 1241 individuals who had no observable retinopathy or had mild retinopathy (DR0 or DR1) at baseline were included. Of these, 209 individuals progressed to more severe retinopathy (grade DR2 or higher) in the 3 year follow-up period. In the same cohort there were 432 participants who died prior to progression, and 600 individuals who were censored.

The hazards of developing moderate or more severe retinopathy were 1.96, 2.71 and 2.82 for Lp-PLA_2_ activity quartiles 2, 3 and 4, respectively, compared with quartile 1 (Table 2). The overall omnibus test for association across the quartiles of Lp-PLA_2_ activity was highly significant (*p* = 1.5 × 10^−5^) and independent of traditional risk factors for diabetic retinopathy. This analysis was adjusted for diabetes-controlling medication, HbA_1c_ levels, SBP, LDLc levels and the age of the individual and was therefore independent of traditional risk factors.

The accompanying cumulative incidence plot (Fig. 2b) is unadjusted and shows the increased hazards of developing moderate or more severe forms of retinopathy by Lp-PLA_2_ quartiles compared with the lowest quartile (light blue line).

#### Association of Lp-PLA_2_ activity with progression to referable or proliferative diabetic retinopathy from lower grades

For this analysis, the cohort of 1364 individuals who had no observable retinopathy, or had mild or observable retinopathy (DR0, DR1, or DR2) at baseline were included. Of these, 461 individuals progressed to more severe retinopathy (grade DR3 or higher) in the 3 year follow-up period. In the same cohort there were 435 participants who died prior to progression, and 468 individuals who were censored.

The hazards of developing severe retinopathy were 1.64, 1.98 and 1.87 for Lp-PLA_2_ quartiles 2, 3 and 4 respectively compared with quartile 1 (Table 2). The omnibus test of association across the quartiles of Lp-PLA_2_ activity levels was highly significant (*p* = 2.8 × 10^−3^) and independent of traditional risk factors for diabetic retinopathy. This analysis was adjusted for grade at baseline, diabetes-controlling medication, HbA_1c_ levels, SBP, smoking status and the age of the individual and therefore independent of traditional risk factors.

The cumulative incidence plot (Fig. 2c) is unadjusted and shows the increased hazards of progressing to referable or proliferative retinopathy from lower grades by Lp-PLA_2_ quartiles compared with the lowest quartile (light blue line).

ESM Table 2 shows HRs for Lp-PLA_2_ quartiles for each analysis with every level of adjustment.

### Sensitivity analyses for extended follow-up period

When considering the longer follow-up period of 5 years, the association with Lp-PLA_2_ activity with incident diabetic retinopathy was attenuated (HR 1.17, 1.14 and 1.26 for Lp-PLA_2_ quartiles 2, 3 and 4 respectively) with only the highest quartile remaining significant (*p* = 0.03). Further details are provided in ESM Table 3 and ESM Fig. 2. Therefore, it was decided to limit our analysis to a 3 year follow-up period.

## Discussion

As a result of the association of Lp-PLA_2_ with increased risk for cardiovascular disease [23] we observe that high Lp-PLA_2_ activity levels are prospectively associated with an increased risk of death in this cohort of individuals with type 2 diabetes. All analyses for retinopathy outcomes were therefore adjusted for this factor by using a competing risk analysis, which directly estimates the effect of Lp-PLA_2_ activity separately on the event of interest (incidence or progression) and the competing event (death), and also allows non-informative censoring for participants when neither event occurred before the end of the study period.

Lp-PLA_2_ activity was an independent predictor for development of incident diabetic retinopathy. Analysing progression to any grade resulted in a similar finding. The association of baseline Lp-PLA_2_ activity with incidence and progression of diabetic retinopathy was highly statistically significant and was independent of LDLc and other traditional risk factors including HbA_1c_, blood pressure, lipid-controlling medication, diabetes-controlling medication, age and sex. A progressive trend of risk by Lp-PLA_2_ activity quartiles can be observed in the first two analyses (incident diabetic retinopathy and progression to observable or more severe grades), where every subsequent quartile confers additional risk to the development of retinopathy or the progression to more severe stages.

Previously, well-established risk factors for diabetic retinopathy included duration of diabetes and poor glycaemic control and blood pressure. This study presents evidence that a proinflammatory enzyme is associated with both the incidence of diabetic retinopathy, as well as progression to more severe stages. Crucially, this is a potential therapeutic target, for which existing antagonists could be repurposed.

The conclusions of these results are supported by the Bradford Hill causal criteria for epidemiological studies [32]: the associations are robust (statistically significant and do not change drastically with the addition of covariates), consistent (across all analyses, albeit not externally validated) and show specificity to Lp-PLA_2_ in models adjusted for potential confounders. Additionally, the results are temporally valid since the measurement of exposure (Lp-PLA_2_) precedes the outcome (retinopathy) and the association is sensitive to the time between the two, with attenuation on increasing time from the date of the activity measure (it is easier to predict the immediate future). The association shows a biological gradient (higher quartiles of Lp-PLA_2_ levels are associated with increased hazards of incidence and progression). There is a plausible mechanism for the association as being due to vascular leakage, and experimental evidence (from animal studies) supports the hypothesis that inhibition of Lp-PLA_2_ results in reduced leakage across the blood–retinal and blood–brain barriers [24–26]. The findings of this study will have to be replicated externally in other cohorts.

Limitations of this study include the fact that serum was collected at a random point in the disease progression for each individual, so it was not consistent by disease state. In addition, 10% of the cohort had proliferative retinopathy at the time of serum collection, hence data from these participants were uninformative in a prospective analysis of incidence. A further 70% of the full cohort had inadequate follow-up for the main analyses, with neither death nor retinopathy assessment in 3 years after baseline. However, this would be an interesting area for exploration, given the evidence presented here. Sensitivity analyses using longer follow-up periods benefited from larger sample size (fewer participants with inadequate follow-up) but potentially introduced other biases, such as increased competing risks and a longer gap between measurement of Lp-PLA_2_ and occurrence of outcome. It is possible that the association between Lp-PLA_2_ activity and onset or progression of diabetic retinopathy is confounded (by factors unknown or unmeasured at baseline) or is biased (e.g. by another unidentified competing risk, or by another mechanism of informative censoring). The nature of an observational epidemiological study means that it is impossible to rule out the possibility of such confounding or biases.

However, complete records of retinopathy screening data for all individuals in a large cohort of individuals with diabetes is a powerful resource. A further strength is the rich set of clinical and questionnaire baseline data that can be used to adjust for known or suspected confounders, and outcome data that can be used to adjust for competing risks.

### Interpretation

Lp-PLA_2_ activity levels are shown to be associated with an individual’s risk of developing diabetic retinopathy. Individuals with Lp-PLA_2_ activity levels above the median have a very high risk of progressing to more severe stages. Since this relationship appears to be independent of traditional risk factors and, specifically, independent of baseline LDLc levels, these data are consistent with the hypothesis that systemic inhibition of Lp-PLA_2_ activity may be a good therapeutic target in the prevention of this complication of diabetes.

### Application

This is a population cohort of white Scottish individuals, with many variables captured using routinely collected health record information. The study population is representative of the diabetic population in Scotland undergoing routine screening [10]. The duration of type 2 diabetes is an important risk factor when considering the extrapolation of these results; diabetic retinopathy is more prevalent when duration of type 2 diabetes is longer than 10 years [2]. This is reflected in the duration of type 2 diabetes in the study population, which, with the addition of 3 years of follow-up, is on average 12 years. Furthermore, the rate of adherence to annual, recommended screens for those with pre-existing retinopathy was reported to be as low as 61%, while the rate of screening for those with long duration of type 2 diabetes was 57% [33]. Overall there is evidence to suggest that adherence to annual screens range between 34% and 65% [3, 9, 33–35]. This suggests that the drop-off rate noted with the 3 year follow-up exclusion criteria is reflective of real-world consumption of healthcare in a population with type 2 diabetes. Further, association characteristics of Lp-PLA_2_ activity are similar to those reported elsewhere [21–23]. Therefore the generalisability of the study to a population of European descent with routine access to healthcare is likely to be high. On the basis of previous observations in animal and human studies it is unlikely that the ethnicity of the cohort would limit the generalisability of this study.

## Electronic supplementary material

ESM (PDF 701 kb)

## Data Availability

Data is available through application to the GoDARTS data acquisition committee.

## Abbreviations

DARTS: Diabetes Audit and Research in Tayside Scotland
DBP: Diastolic blood pressure
DME: Diabetic macular oedema
DR(0–4): Diabetic retinopathy (grade)
GoDARTS: Genetics of Diabetes Audit and Research in Tayside Scotland
LDLc: LDL-cholesterol
Lp-PLA_2_: Lipoprotein-associated phospholipase A_2_
SBP: Systolic blood pressure
