# Supplementary material for: Lp-PLA2 activity is associated with increased risk of diabetic retinopathy: a longitudinal disease progression study
Source: Diabetologia. 2018 Apr 6;61(6):1344–53. doi: 10.1007/s00125-018-4601-7 (PMC6447502; doi:10.1007/s00125-018-4601-7)
Supplement: Supplementary file 1 — (PDF 701 kb) [file 125_2018_4601_MOESM1_ESM.pdf]

Online-Only Supplemental Material

Title: Lp-PLA_2 activity is associated with increased risk of diabetic retinopathy: a longitudinal disease progression study

Moneeza K Siddiqui [1], Gwen Kennedy [2], Fiona Carr[1], Alexander SF Doney [2], Ewan Pearson [1], Andrew D Morris [3], Toby Johnson [4], Megan M McLaughlin [5], Rachel E Williams [6], Colin NA Palmer [1]

Author Affiliations

[1] Pat McPherson Centre for Pharmacogenetics and Pharmacogenomics, University of Dundee School of Medicine, United Kingdom

[2] Ninewells Hospital and Medical School, University of Dundee, United Kingdom

[3] Usher Institute of Population Health Sciences and Informatics, University of Edinburgh, United Kingdom

[4] GlaxoSmithKline Medicines Research Centre, Gunnels Wood Road, Stevenage, United Kingdom

[5] Alternative Discovery and Development, Research & Development, GlaxoSmithKline, King of Prussia, PA

[6] Worldwide Epidemiology, GlaxoSmithKline, Collegeville, PA

Running head: Lp-PLA_2 activity and diabetic retinopathy

Address for reprints

Professor Colin N A Palmer ORCID: 0000-0002-6415-6560
Pat McPherson Centre for Pharmacogenetics and Pharmacogenomics
University of Dundee School of Medicine
Ninewells Hospital and Medical School
United Kingdom
Telephone: +44 01382 383155
Fax: +44 01382 383598
Email: c.n.a.palmer@dundee.ac.uk

Competing Risk: Association of Lp-PLA_2 and death in the GoDARTS cohort

ESM Table 1: Hazards ratios of death by Lp-PLA_2 quartiles in the GoDARTS cohort, adjusted for gender and age

**Lp-PLA2 quartiles**	**Hazard Ratios (95% CI)**	***P* value**
1	--	--
2	0.98 (0.84, 1.14)	0.77
3	1.26 (1.09, 1.46) **	0.002
4	1.45 (1.24, 1.68) ***	<0.0001

ESM Table 2. Hazard Ratios of Lp-PLA$_2$ quartiles in all model shown separately for each variable added to develop the final model

Variables in model	Hazard Ratios (95% Confidence Limits) of Lp-PLA$_2$ quartiles	*P*-values
**Development of incident DR from disease-free state (DR0 to DR1 or higher) n = 1013**		
Lp-PLA$_2$ quartiles		
Lp-PLA$_2$ Quartile 2 v. 1	1.33 (1.08, 1.64)	0.0079
Lp-PLA$_2$ Quartile 3 v. 1	1.56 (1.28, 1.90)	<0.0001
Lp-PLA$_2$ Quartile 4 v. 1	1.52 (1.24, 1.86)	<0.0001
Lp-PLA$_2$ quartiles + sex		
Lp-PLA$_2$ Quartile 2 v. 1	1.37 (1.11, 1.68)	0.0033
Lp-PLA$_2$ Quartile 3 v. 1	1.60 (1.31, 1.97)	<0.0001
Lp-PLA$_2$ Quartile 4 v. 1	1.60 (1.31, 1.96)	<0.0001
Lp-PLA$_2$ quartiles + sex + diabetic medication		
Lp-PLA$_2$ Quartile 2 v. 1	1.38 (1.12, 1.70)	0.0024
Lp-PLA$_2$ Quartile 3 v. 1	1.58 (1.30, 1.93)	<0.0001
Lp-PLA$_2$ Quartile 4 v. 1	1.61 (1.32, 1.97)	<0.0001
Lp-PLA$_2$ quartiles + sex + diabetic medication + use of statins		
Lp-PLA$_2$ Quartile 2 v. 1	1.41 (1.14, 1.74)	0.0014
Lp-PLA$_2$ Quartile 3 v. 1	1.63 (1.33, 1.98)	<0.0001
Lp-PLA$_2$ Quartile 4 v. 1	1.71 (1.40, 2.10)	<0.0001
Lp-PLA$_2$ quartiles + sex + diabetic medication + use of statins + HbA$_{1c}$		
Lp-PLA$_2$ Quartile 2 v. 1	1.35 (1.08, 1.67)	0.0072
Lp-PLA$_2$ Quartile 3 v. 1	1.56 (1.27, 1.91)	<0.0001
Lp-PLA$_2$ Quartile 4 v. 1	1.70 (1.38, 2.09)	<0.0001
Lp-PLA$_2$ quartiles + sex + diabetic medication + use of statins + HbA$_{1c}$ + SBP		
Lp-PLA$_2$ Quartile 2 v. 1	1.35 (1.08, 1.67)	0.0075
Lp-PLA$_2$ Quartile 3 v. 1	1.53 (1.24, 1.88)	<0.0001
Lp-PLA$_2$ Quartile 4 v. 1	1.69 (1.37, 2.08)	<0.0001
Lp-PLA$_2$ quartiles + sex + diabetic medication + use of statins + HbA$_{1c}$ + SBP + LDLc		
Lp-PLA$_2$ Quartile 2 v. 1	1.52 (1.21, 1.91)	0.0004
Lp-PLA$_2$ Quartile 3 v. 1	1.71 (1.37, 2.15)	<0.0001
Lp-PLA$_2$ Quartile 4 v. 1	2.08 (1.64, 2.64)	<0.0001
Lp-PLA$_2$ quartiles + sex + diabetic medication + use of statins + HbA$_{1c}$ + SBP + LDLc + age		
Lp-PLA$_2$ Quartile 2 v. 1	1.52 (1.21, 1.91)	0.0003
Lp-PLA$_2$ Quartile 3 v. 1	1.72 (1.37, 2.16)	<0.0001
Lp-PLA$_2$ Quartile 4 v. 1	2.08 (1.64, 2.63)t	<0.0001
**Progression to observable or more severe DR from lower grades (DR0 or DR1 to DR2 or higher) n = 1241**		
Lp-PLA$_2$ quartiles		
Lp-PLA$_2$ Quartile 2 v. 1	1.86 (1.29, 2.77)	0.0023
Lp-PLA$_2$ Quartile 3 v. 1	2.33 (1.59, 3.42)	<0.001
Lp-PLA$_2$ Quartile 4 v. 1	2.21 (1.46, 3.33)	0.0002
Lp-PLA$_2$ quartiles + HbA$_{1c}$		
Lp-PLA$_2$ Quartile 2 v. 1	1.69 (1.12, 2.55)	0.012
Lp-PLA$_2$ Quartile 3 v. 1	2.11 (1.41, 3.17)	0.0003
Lp-PLA$_2$ Quartile 4 v. 1	1.96 (1.27, 3.01)	0.002
Lp-PLA$_2$ quartiles + HbA$_{1c}$ + SBP		

Lp-PLA_2 Quartile 2 v. 1	1.70 (1.13, 2.56)	0.012
Lp-PLA_2 Quartile 3 v. 1	2.11 (1.41, 3.17)	0.0003
Lp-PLA_2 Quartile 4 v. 1	1.97 (1.28, 3.02)	0.002
Lp-PLA_2 quartiles + HbA_{1c} + SBP + LDLc		
Lp-PLA_2 Quartile 2 v. 1	1.91 (1.23, 2.99)	0.004
Lp-PLA_2 Quartile 3 v. 1	2.52 (1.64, 3.88)	<0.0001
Lp-PLA_2 Quartile 4 v. 1	2.43 (1.48, 3.97)	0.0004
Lp-PLA_2 quartiles + HbA_{1c} + SBP + LDLc + age		
Lp-PLA_2 Quartile 2 v. 1	1.91 (1.22, 2.99)	0.0045
Lp-PLA_2 Quartile 3 v. 1	2.62 (1.70, 4.04)	<0.0001
Lp-PLA_2 Quartile 4 v. 1	2.79 (1.70, 4.59)	<0.0001
**Progression to referable or proliferative DR from lower grades (DR0, DR1 or DR2 to DR3 or higher) n = 1364**		
Lp-PLA_2 quartiles		
Lp-PLA_2 Quartile 2 v. 1	1.76 (1.23, 2.50)	0.0018
Lp-PLA_2 Quartile 3 v. 1	1.81 (1.26, 2.60)	0.0013
Lp-PLA_2 Quartile 4 v. 1	1.83 (1.25, 2.66)	0.0017
Lp-PLA_2 quartiles + DR grade at baseline		
Lp-PLA_2 Quartile 2 v. 1	1.83 (1.28, 2.60)	0.0008
Lp-PLA_2 Quartile 3 v. 1	1.89 (1.31, 2.73)	0.0006
Lp-PLA_2 Quartile 4 v. 1	1.97 (1.35, 2.86)	0.0004
Lp-PLA_2 quartiles + DR grade at baseline + use of diabetic medication		
Lp-PLA_2 Quartile 2 v. 1	1.82 (1.28, 2.60)	0.0009
Lp-PLA_2 Quartile 3 v. 1	1.96 (1.36, 2.83)	0.0003
Lp-PLA_2 Quartile 4 v. 1	2.06 (1.42, 3.00)	0.0001
Lp-PLA_2 quartiles + DR grade at baseline + use of diabetic medication + HbA_{1c}		
Lp-PLA_2 Quartile 2 v. 1	1.70 (1.19, 2.45)	0.004
Lp-PLA_2 Quartile 3 v. 1	1.88 (1.28, 2.76)	0.0014
Lp-PLA_2 Quartile 4 v. 1	1.85 (1.26, 2.72)	0.0018
Lp-PLA_2 quartiles + DR grade at baseline + use of diabetic medication + HbA_{1c} + SBP		
Lp-PLA_2 Quartile 2 v. 1	1.70 (1.18, 2.44)	0.004
Lp-PLA_2 Quartile 3 v. 1	1.87 (1.27, 2.74)	0.0015
Lp-PLA_2 Quartile 4 v. 1	1.85 (1.25, 2.72)	0.002
Lp-PLA_2 quartiles + DR grade at baseline + use of diabetic medication + HbA_{1c} + SBP + smoking status		
Lp-PLA_2 Quartile 2 v. 1	1.74 (1.21, 2.50)	0.0027
Lp-PLA_2 Quartile 3 v. 1	2.02 (1.36, 2.98)	0.0004
Lp-PLA_2 Quartile 4 v. 1	1.97 (1.33, 2.93)	0.0008
Lp-PLA_2 quartiles + DR grade at baseline + use of diabetic medication + HbA_{1c} + SBP + smoking status + age		
Lp-PLA_2 Quartile 2 v. 1	1.75 (1.22, 2.52)	0.0024
Lp-PLA_2 Quartile 3 v. 1	2.06 (1.40, 3.04)	0.0003
Lp-PLA_2 Quartile 4 v. 1	1.99 (1.34, 2.94)	0.0006

Sensitivity analysis – 5 year follow-up period

N = 2452, number of incident DR = 1038, number of competing events = 330, number censored = 1084

ESM Table 3 Hazard ratios for quartiles of Lp-PLa$_2$ at 5 years in a 5 year follow-up period

Lp-PLA$_2$ quartiles	Hazard Ratios (95% CI)	*P* value
**Main effects model**		
Q1	--	--
Q2	1.12 (0.94, 1.35)	0.21
Q3	1.18 (0.99, 1.40)	0.06
Q4	1.15 (0.97, 1.37)	0.11
**Adjusted model** (adjusted for use of diabetic medication, lipid modifying medication, HbA1c, SBP, LDL, sex, and age)		
Q1	--	--
Q2	1.17 (0.95, 1.43)	0.13
Q3	1.14 (0.93, 1.40)	0.20
Q4	1.26 (1.02, 1.56)	0.03

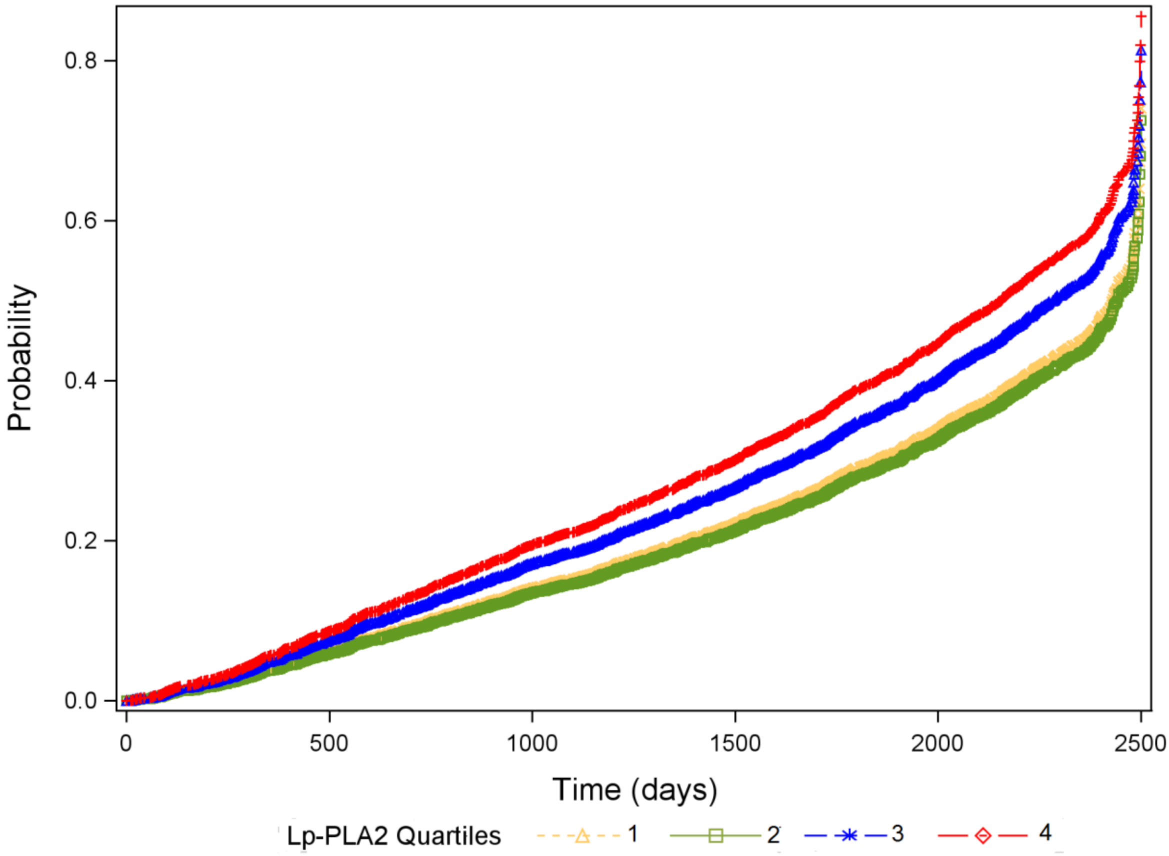

ESM Fig 1 Survival Analysis – probability of death by Lp-PLA$_2$ quartiles in the GoDARTS cohort (n = 6371). The model was adjusted for gender and age

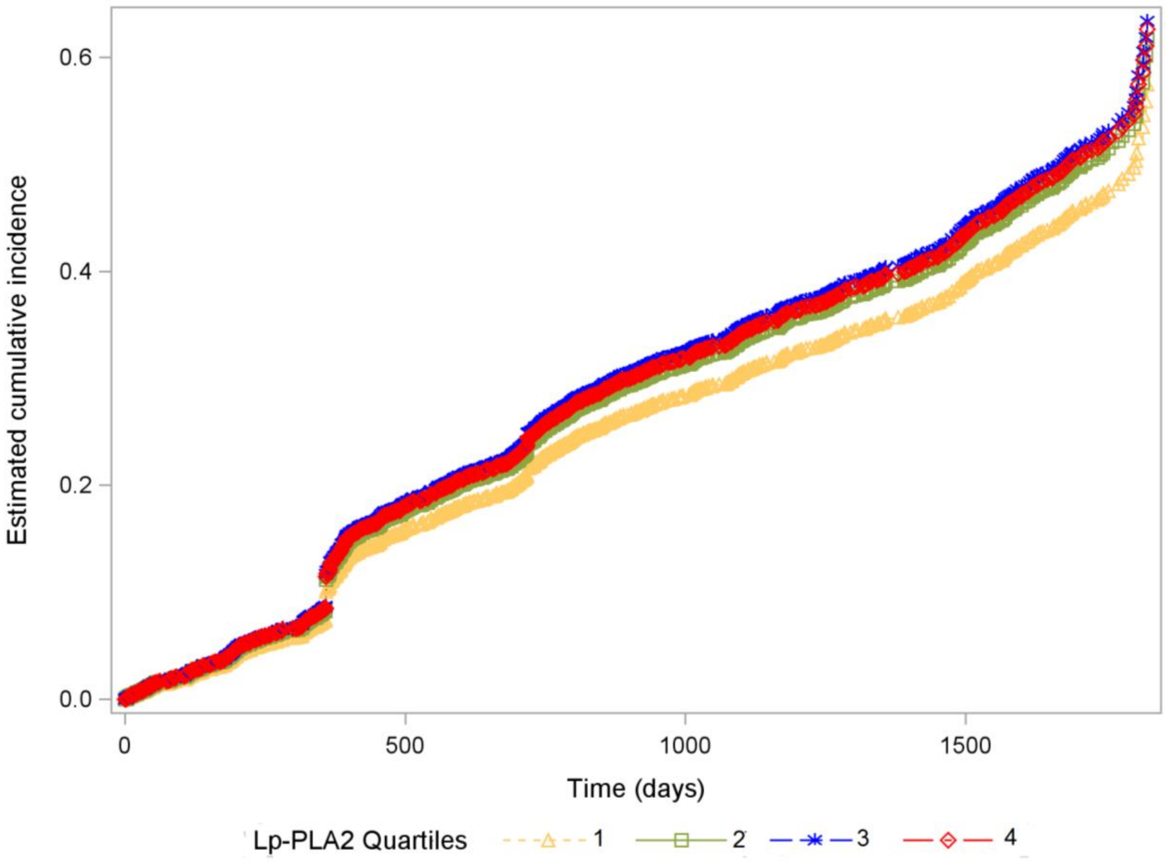

ESM Fig 2 Cumulative incidence plot for the main effect of Lp-PLA$_2$ quartiles on the development of incident DR adjusted for the competing risk of death in a 5 year follow-up period.
